# Teachers' Value Consonance and Employee-Based Brand Equity: The Mediating Role of Belongingness and Self-Efficacy

**DOI:** 10.3389/fpsyg.2022.900972

**Published:** 2022-05-26

**Authors:** Xianbi Yang, Abrar Hussain Qureshi, Yenku Kuo, Nguyen Ngoc Quynh, Tribhuwan Kumar, Worakamol Wisetsri

**Affiliations:** ^1^Institute of Education, Xiamen University, Xiamen, China; ^2^Hangzhou Normal University Qianjiang College, Hangzhou, China; ^3^Department of English, University of Sahiwal, Sahiwal, Pakistan; ^4^Bachelor Program in Leisure Industry Management, Commercial College, Chinese Culture University, Taipei City, Taiwan; ^5^Department of Economics, Thuong Mai University, Hanoi, Vietnam; ^6^College of Science and Humanities at Sulail, Prince Sattam bin Abdulaziz University, Al Kharj, Saudi Arabia; ^7^Department of Manufacturing and Service Industry Management, Faculty of Business and Industrial Development, King Mongkut's University of Technology North Bangkok, Bangkok, Thailand

**Keywords:** value consonance, teachers self-efficacy, belongingness, employee brand based equity, employee behavior and attitudes

## Abstract

This study investigated the impact of value consonance on employee-based brand equity through the mediating role of teachers' self-efficacy and belongingness. For this purpose, a deductive approach was followed, and data were collected under a cross-sectional research design from academia through a questionnaire. Prior approval from the administration was sought before administrating the questionnaire on a large scale and a sample of 520 teachers was approached in the first phase. At this stage, 418 answered questionnaires were received, while in the second wave, questions related to the teacher's self-efficacy and employee-based brand equity were asked from the respondents. Out of these 418 re-distributed questionnaires, 387 were received back and after discarding the partially filled and incomplete questionnaires, the useable sample size was left as 372. Data have been analyzed by using the structural equation modeling technique, which was assessed through measurement and structural model. Results indicate that value consonance can promote positive behaviors in the workplace. Moreover, teachers with high self-efficacy can develop based on brand equity. Similarly, employees with high-value consonance develop a sense of belongingness with their academic institutes. Limitations and future directions are also discussed.

## Introduction

Branding has got new dimensions and due to its competitive nature, it has become the most sought-after commodity in organizations (Urde, 2016). (Backhaus and Tikoo, 2004) claimed that branding isn't just about things or commodities; it is more about people. Additionally, branding was originally utilized to distinguish between services and goods, but it evolved through time to be connected to individuals, allowing corporations to differentiate themselves from competitors. Internal branding is a multi-dimensional approach that is used to promote an organization's brand among its personnel (Biswas). According to several experts, brand characteristics are transferred from corporations to consumers *via* employees who interact with consumers' requirements (King and Grace, 2008).

Seemingly, many company employees utilize internal branding for engaging new employees and sending out important thoughts about the organization. In the service industry, brand equity has received special attention. According to an example (Berry, 2000), service BE is established by successful brand service and customer experiences, based on the development of a service-branding paradigm. Interestingly, there are several inconsistencies among the various systems often used to measure based brand equity, because they do not concur within the same parameters and, therefore, do not fully emphasize the determinant factors and the parameters of based brand equity in the service business (Mourad et al., 2020).

In reaction to global student mobility and declining institutional finance, higher education institutions have been forced to compete for student enrollment. Higher education institutions have been obliged to implement branding and corporate brand initiatives as market demands have increased (Palmer et al., 2016). As a result of these developments, the primary goal appears to be to concentrate on brand construction that is based on two-way interaction within the branding strategy while also acknowledging the contributions of institutions and faculties to branding. Because a great institutional brand has the power to capture clients' trust and loyalty, the expenditure on branding educational institutions has increased dramatically (Pinar et al., 2014).

However, regarding an argument that higher education and branding go back a long way, the literature on branding in higher education is scant. The notion that branding in the non-profit sector promotes unhealthy rivalry and is a waste of resources is debunked by the awareness that established companies are strategic assets in the industry that create brand value. Even though educational institutes are a mix of locations, classes, programs, and certifications, branding has now become critical in managing this service (Retamosa et al., 2020). Personal values relating to one's activities, as well as environmental influences, are significant components that give inspiration and judgment calls.

Quite recently, a study has found that, in conjunction with one's convictions, how these values coincide with the perceived values espoused by one's employer affects professional wellbeing and perseverance (Edwards and Cable, 2009). Such perceived fit among personal and corporate values, known as value consonance, is increasingly being studied as a socio-cultural measure of wellbeing in work situations (Edwards and Cable, 2009). Perceived consonance between someone's values and those of someone's employer has been shown to have a favorable effect on perseverance and psychological wellbeing, as evidenced by increased employee satisfaction, organizational identity, effective communication, and inclination to continue (Edwards and Cable, 2009).

Increased levels of consonance among teachers' values and that of their campus community (i.e., estimated scores throughout teaching staff at their college/university) have been linked to greater job satisfaction, higher work commitment, reduced exhaustion in the teaching profession, and is also likely related to communicated teaching exuberance in the classroom (Wang and Hall, 2019). There are two types of self-efficacy mentioned in reported literature on educational contexts, i.e., teachers' self-efficacy and collective teachers' self-efficacy (Skaalvik and Skaalvik, 2019). Teachers' self-efficacy, or instructors' willingness and ability to plan and carry out the actions necessary to achieve specific goals, has garnered considerable attention in educational research over the last few decades.

Teacher self-efficacy is linked to several good outcomes, including better levels of teacher engagement and work satisfaction, as well as reduced levels of stress and burnout (Skaalvik and Skaalvik, 2019). Teachers' self-efficacy is a powerful tool for evaluating the well-being of the teachers and job satisfaction has been explored well (Barni et al., 2019). Several types of research have shown the importance to investigate the relationship between different aspects of professional competence, instructional behavior, and academic achievement gains. However, just a few studies have looked into the relationship between teachers' tendencies and their professional commitment (Skaalvik and Skaalvik, 2019), particularly in terms of building brand equity among themselves as employee-based brand equity (EBBE).

Situational discord or consonance can have major consequences on a person's relationship with his or her surroundings (Rosenberg, 1977). Cognitive dissonance can lead to a sense of someone not belonging, a sense that someone does not belong, that one is out of place, and that one is doing something wrong. Sense of belonging is defined, according to one study (Cueto et al., 2009), as the degree to which people feel socially attached. The desire to be a part of something larger than oneself is a universal human desire. Belongingness, or the sense of belonging, encompasses the urge to be recognized and associated with a specific group. The desire to belong to a group can be found among school pupils, colleagues, a sporting team, a teaching team, or a community group. A sense of belonging extends beyond knowing exactly or being acknowledged by others. Gaining recognition, acceptance, and encouragement from group members, as well as doing the same, on the other hand, are the main motivations of belongingness (Cueto et al., 2009).

Learners' sense of belonging is defined as “the extent to which they feel connected with their classmates and teachers at educational institutes. Teachers' belongingness at the institute can be described as the extent to which they experience being integrated with institutions, authorities, and learners, based on this perspective. Furthermore, belongingness, according to one study (Goodenow, 1993), necessitates feelings of being accepted, appreciated, and cherished. Additionally, teachers who are apprehensive about reflecting ideas that are incompatible with their values may face cognitive dissonance, making teaching and class administration uncomfortable. An individual in a consonant setting, on the other hand, seems to be more likely to feel like they belong (Skaalvik and Skaalvik, 2019).

This based brand equity has been utilized in different service sectors and as part of the service sector, higher education institutes could also be evaluated for their based brand equity (Mourad et al., 2020). The findings of (Mourad et al., 2020)'s research suggested further exploration of based brand equity in the context of higher educational institutes. Keeping in mind the role of teachers as employees in educational institutes could lead to employee-based brand equity. No such research has been carried out in past for evaluating the teachers' value consonance in developing based brand equity so to bridge this gap in previous investigations, this study is providing a novel integration of the supposed connections.

As discussed by Barni et al. (2019), teachers' self-efficacy could lead to teachers' wellbeing and job satisfaction, we assume that it could also help in developing based brand equity in educational institutes while being utilized as a mediator, therefore mediating the impact of teachers' self-efficacy was also evaluated between teachers' value consonance and based brand equity. As suggested by Skaalvik and Skaalvik (2019), the belongingness of teachers is connected with their value consonance so, we utilized it as a mediator as well which could further help in strengthening based brand equity. This research would answer certain questions about the association of teachers' value consonance with based brand equity with the help of teachers' self-efficacy and belongingness.

## Theoretical Support

“Efficacy beliefs influence how environmental possibilities and barriers are evaluated,” according to social cognition theory (Lee et al., 2019). As a result, people's objectives, motivation, and behaviors are influenced by their self-efficacy. Teacher self-efficacy is linked to better levels of teacher engagement, work satisfaction, and dedication, as well as relatively low levels of depression and desire to quit the profession, according to research (Moyano et al., 2021). Individuals, according to Bandura's (1997) social cognitive theory, have a self-structure that lets them exert some control over their ideas, emotions, motivation, as well as behaviors. This self-system includes reference mechanisms as well as a collection of modules for observing, controlling, as well as assessing behavior in any way of the system's engagement with environmental factors that have an impact. As such, it performs a self-regulatory role by allowing individuals to affect their internal cognitive patterns and behaviors, so modifying their surroundings (Diestel, 2021).

A person can maintain influence over whatever he or she performs, according to Bandura's social cognitive theory, but there is a reciprocating link between a person's conduct and the environment, with his or her cognition (Bandura, 1999). Self-efficacy is a component of Bandura's social cognitive theory that changes a person's objectives, behaviors, and actions and is impacted by environmental factors. Self-efficacy is defined by Bandura (1997, p. 3) as “religious views in one's capacities to plan and manage a business venture necessary to accomplish given attainment”. These attitudes, rather than being permanent character qualities, are dynamic and learning structures that are impacted by how people see possibilities and constraints in their surroundings (Preece and Bullingham, 2022).

### Value Consonance and Employee-Based Brand Equity

Brand equity (BE), a valuable marketing asset, generates competitive advantages and boosts an organization's financial success. BE research has mostly focused on the customer perceptions of a product's brand value when exposed to branding and marketing elements (Rauschnabel et al., 2019). BE concepts and indicators are diverse but ambiguous and are frequently being measured by business performance. However, among the many concepts, the concept of BE will often incorporate marketing impacts that are exclusive to a given brand (Holiday et al., 2021). The strength of a brand is determined by customers' convictions and impressions based on what they have learned, felt, and seen, as well as heard (Hussain et al., 2020). Because of the growing interest among researchers in brand management, researchers have expanded the variety of indicators used to quantify brand value beyond purely financial factors (Edeling et al., 2020).

Employees' thoughts and opinions, attitudes, and behaviors toward the brand, which appear externally as brand promises, are gathered through their work experiences and contacts with the firm. Employees' positive and constructive brand behaviors that come from brand awareness of the desired behavior connected to the brand identity are classified as EBBE (Moyano et al., 2022). As a result, EBBE refers to workers' internalization of a brand's fundamental values, as seen by brand image and brand loyalty, as well as brand value congruence (Liu et al., 2020). Employee brand endorsement happens when workers communicate beneficial information to individuals outside the business through word of mouth; employee brand loyalty focuses on employees' intentions to stay with the organization (Gamage and Tajeddini, 2022).

Employee conduct that naturally corresponds to organizational principles without explicit teaching is referred to as brand value consistency (Ozuem et al., 2022). All these actions occur on their own and do not require any prior training. Until now, the majority of the studies on the elements that lead to EBBE have focused on employees' psychological perspectives (Hanaysha and Al-Shaikh, 2021). Value consonance is characterized as teachers' belief that they accept the university's dominant values, as well as norms, and about which aims should have been pursued, which content should have been emphasized, and which educational approaches and procedures could've been used (Stacey, 2022). This shows that value consonance has a positive relationship with employee-based brand equity. And from all this previous research this hypothesis shows a positive relationship between them.

*H*_1_: *Value consonance is positively related to employee-based brand equity*

### Value Consonance and Teachers' Self-Efficacy

Value consonance is characterized as how instructors believe they embrace the university's prevalent values and norms, such as goals that should have been pursued, material that should be stressed, and educational techniques and procedures that should be employed (Stacey, 2022). A teacher who believes in the university's prevailing principles and rules that are contradictory to his or her own beliefs may experience contextual contradiction, whereas a teacher who appreciates the university's prevalent norms and values may suffer contextual consonance (Wernhart et al., 2019). Contextual consonance, as well as dissonance, can emerge in social identity settings, competency contexts, contextual factors, and even cultural contexts (Skaalvik and Skaalvik, 2019).

Under social cognitive theory and social cognitive career theory, self-efficacy has been identified as the most potent self-regulatory tool for influencing work behavior, motives, and target behavior (Bandura, 1997). Therefore, experts have defined the teacher-related component of self-efficacy as a teacher's belief in their ability to successfully execute a given teaching job required to achieve certain goals in a specific situation. As per available literature on this kind of relationship, value consonance has been linked to self-efficacy in high school students, service employees, and office staff (Nangoy, 2018). Some authors (Skaalvik and Skaalvik, 2019) also claimed that value contention (the polar opposite of value consonance) affects teachers' self-efficacy. The instructors' confidence in their talents declines when they believe that the schools' dominant principles are incompatible with their convictions. As a result, we anticipate that value consonance will help in shaping teachers' self-efficacy.

Additionally, Rosenberg defined the value context as the link between a given person's feature and the value assigned to that attribute in the perspective of that individual's surroundings (Rosenberg, 1977). We extend the idea to include the alignment of a teacher's aims, conventions, and attitudes regarding teaching and education, with the goals, social standards, and values that exist, at the university where he or she is employed. Enhancing the learning experiences, vicarious learning, social competence, and physiological and emotional states are all sources of information that influence self-efficacy beliefs (Myyry et al., 2022). As a result, the current work fulfills this need by examining the positive relationship between value consonance and teacher self-efficacy.

*H*_2_: *Value consonance is positively related to teacher self-efficacy*

### Value Consonance and Belongingness

Belonging is defined as a sense of affiliation and worth (Fan et al., 2020). A sense of belonging, according to Fan et al. (2020), is derived from a sense of being accepted, respected, and getting social support from other members of the community. As a result, we predicted teachers' feelings of belonging to be favorably related to pleasant and supportive relationships with colleagues and the university administration (Greenier et al., 2021). We also anticipated that value consonance would be linked to a sense of belonging, as sharing values promotes acceptance and respect. A previous study has found a moderate to the high link between value consonance and teachers' feelings of belonging, which supports this hypothesis (Jensen, 2022).

The desire to belong is an essential human motivator, according to Turk et al. (2022). The degree to which they want to belong is met, for example at work, can have an impact on motivation, dedication, and also well. At the student level, a sense of belonging to the university or a college class is linked to academic motivation and good effect (Marler et al., 2021). A study (Mérida-López et al., 2022) also found that teachers' feelings of belonging at their place of employment were linked to increased work satisfaction and reduced levels of emotional weariness.

Situational disharmony or consonance might have major consequences on a person's interaction with his or her surroundings. Situational dissonance can lead to a sense of someone not belonging, a sense that one does not belong, because one is out of place or he is doing something wrong (Rosenberg, 1977). Additionally, teachers who are worried about reflecting ideas that are incompatible with their values may feel cognitive dissonance, making education and instructional management uncomfortable. An individual in a consonant setting, on the other hand, is much more inclined to feel like he belongs. So, in accord with the supported arguments, it could be suggested that teachers' feelings of belongingness are positively connected to value consonance. As a result, the previous studies support this need by examining the hypothesis given below.

*H*_3_: *Value consonance is positively related to Belongingness*

### Mediating the Relationship Between Value Consonance and Employee-Based Brand Equity

The teacher's self-efficacy component of social cognition theory has been thoroughly examined in a variety of disciplines and situations, and it has gained support from a substantial number of data from a variety of domains (Fryer et al., 2021). Teachers necessarily communicate, as well as reflect ideas, in their regular teaching and classroom management. The goal of this study was to investigate the relationships between teachers' perceptions of university-level values represented by the university's goal structure, as well as value consonance (the extent to which they felt they decided to share the university's prevailing principles and rules), teachers' feelings of belonging, perceived stress, EBBE, and work satisfaction, as well as motivation to end up leaving the teaching profession (Howe et al., 2019).

Belonging is placed in the center of the pyramid of the motivational hierarchy. It is a requirement, as one study (Wernhart et al., 2019) showed out, as well as its absence has severe consequences. Belongingness is connected to several topics, such as directors' attitudes and behaviors toward employees, organizational styles and communication levels of all employees in the workplace, the degree to which socially and economically expectations for work are met, and the working conditions of the employees (Lamm et al., 2015). It differs from commitment because commitment indicates a one-sided connection, whereas belongingness suggests a two-way interaction (Gamage and Tajeddini, 2022).

Teachers' self-efficacy and belongingness mediate the relationship between value consonance and employee-based brand equity. An employee's feeling of belonging influences their actions and attitudes about work-life, and has a favorable impact on their overall performance (Etehadi and Karatepe, 2019). Belongingness is significant because the more an employee understands a workplace's goals and values, as well as thinks he belongs to the organization or institution where he works, the more driven he is to achieve organizational goals and work for the organization.

Employer branding is a multidimensional technique that organizations may use to attract and recruit skilled personnel. Employer branding may be used as a strategic opportunity to improve employee engagement inside a global market when a competent workforce is rare and has many alternatives (Ibrahim et al., 2018). The main concept behind this assumption is that university employees are aware of the university's requirements and pressure, which helps to improve collaboration (Morawska-Jancelewicz, 2021). As a result, the current study investigates this gap by looking at the role of teachers' self-efficacy and belongingness in mediating the link between value consonance and employee-based brand equity.

*H*_4_ *: Teachers' self-efficacy mediates the relationship between Value consonance and employee-based brand equity*
*H*_5_ *: Belongingness mediates the relationship between Value consonance and employee-based brand equity*

**Figure 1:**
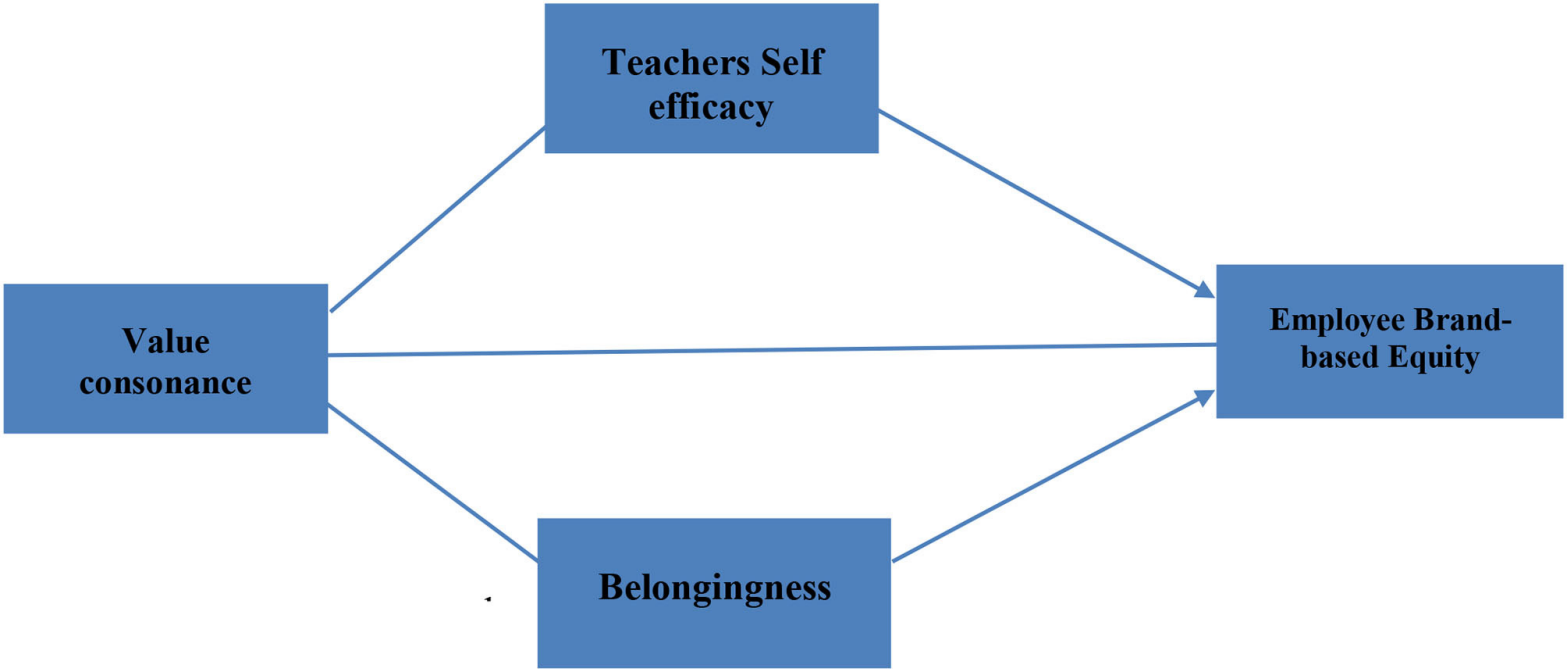
Conceptual framework.

The following conceptual model (Figure 1) has been formed based on the above literature and hypotheses.

## Participants and Procedure

This study followed a deductive approach and data were collected under a cross-sectional research design. Cross-sectional research design is prevalent in social sciences and was most suitable in this study because this study aimed to quantify the impact of value consonance on employee-based brand equity. Thus, in this study, respondents (teachers) from academia were requested to complete a survey questionnaire. Previous studies have documented the perception of teachers while investigating consonance and self-efficacy (Abun et al., 2021). Prior approval from the administration was sought before administrating the questionnaire on large scale. In addition to this, written informed consent was also obtained from the respondents before handing over the questionnaires.

A list of faculty/teaching staff was obtained from the administration and teachers were approached as per the available list in their duty time. It was ensured to them that this study is only for academic purposes and their response will be kept confidential. As a goodwill gesture, a ‘thank you note was served to the participants to boost their motivation to complete the questionnaire.

Before administrating questionnaires, suitable sample size was set based on the available literature and recommendations from the past studies. In this regard, the most used criteria of Krejcie and Morgan (1970) and previously used by Bashir et al. (2019, 2020) and Wu et al. (2022).

Hence, a sample of 520 respondents was devised at the initial stage, and questionnaires were distributed among the teachers in the first phase. At this stage, questions related to demographic characteristics, value consonance (independent variable), and belongingness (mediating variable) were asked from the respondents. A secret code was allotted to each questionnaire so that in the next wave, relevant respondents could be identified easily. At this stage, 418 questionnaires were received back. In the second wave questions related to the teacher self-efficacy (mediating variable) and employee-based brand equity (outcome variable). Out of these 420 re-distributed questionnaires, 387 questionnaires were received back. After discarding the partially filled and incomplete questionnaires, the useable sample size was left as 372 and these responses have been used for final data analysis.

Collecting data in two waves has helped us to overcome the issue of common method biases (Podsakoff, 2003; Malhotra et al., 2006). Moreover, we have employed other techniques, such as using reverse coded questions to restrict the respondents from providing monotonic responses. In addition to this changing the position of variables in questionnaires (through two waves), helped to restrict the respondents from developing a correlation among the study constructs. Demographic characteristics of the respondents were obtained, and it was observed that most of the respondents were i.e., 71%, while female participants were 29%. In the case of qualification, most of the participants have 18 years of education while respondents with ages more than 30 years were high. Similarly, teachers having experience of >5 years were also greater in numbers as compared to the teachers having experience of fewer than 5 years.

### Scales/Measurement

This study followed a 5-point Likert scale to get responses from the participants, where 5 indicates strongly agree and 1 indicates strongly disagree. Value consonance is operationalized based on three items scale developed by Skaalvik and Skaalvik (2011). Sample items for this scale include, “My colleagues and I have the same opinion about what is important in education”. Similarly, the first mediating variable of this study, i.e., teacher self-efficacy is measured based on three dimensions, namely, self-efficacy for instructional strategies and self-efficacy for student engagement. Each dimension was assessed based on four items. This scale is developed by Klassen et al. (2009), sample items for this scale include, “How much can you do to craft good questions for students?”. The language of items of this scale was partially modified to fit it in the context of this study. The second mediating variable of this study, i.e., Belongingness is measured based on three items scale developed by Skaalvik and Skaalvik (2011). Sample items for this scale include, “I feel that I am accepted by the university leadership”. The outcome variable of this study (employee-based brand equity) is measured based on five items scale developed by Baumgarth and Schmidt (2010). This sample item includes, “I am aware that everything I say or do can affect the brand image”.

## Results

### Assessment of Measurement and Structural Model

This study used a multivariate data analysis tool by applying structural equation modeling (SEM) based on partial least square (PLS) SEM methodology. For this purpose, Smart PLS 3.9 was used (Ali et al., 2018; Bashir et al., 2020). Smart PLS provides numerous benefits/advantages; firstly, it deals very comfortable with the complex models and it can handle small samples very efficiently (Hair et al., 2017). Additionally, it can deal with the non-normal data very comfortably and non-parametric data is easily handled by Smart PLS lastly it is used when the theory is less developed. In our case where the theory about employee-based brand equity is less developed, so using Smart PLS was the best option. SEM is assessed in two ways, one way is concerned with the assessment of the measurement model, while another portion is related to the assessment of the structural model (Hair et al., 2012,Hair et al., 2019). Table 1 shows the main results.

**Table 1:** Discriminant validity (Fornell-Larker-1981 Criteria).

**Construct**	**BL**	**EBBE**	**TSE**	**VC**
BL	0.813			
EBBE	0.441	0.737		
TSE	0.288	0.664	0.773	
VC	0.345	0.491	0.300	0.877

*VC, Value consonance; EBBE, Employee based brand equity; TSE, Teacher self-efficacy; BL, Belongingness. The bold values indicate the results for corresponding statistics for whole variable not the items*.

Assessment of the measurement model was done based on reliability and validity. Reliability has been tested based on three parameters, namely, alpha, rho-a, and composite reliability and it has been observed that all the three indicators of reliability are within the acceptable range i.e.,>0.60. For instance, the value of alpha for belongingness is 0.744, for employee-based brand equity is 0.73, for teachers' self-efficacy it is 0.92, and for value consonance, it is 0.855. Thus, the value of alpha for all constructs was above the threshold limit. Similarly, values of composite reliability were also above the prescribed limit. In the case of rho-A values were within the range of 0.76 to 0.93. Hence, all the parameters related to reliability statistics were within the acceptable range and confirmed the reliability of the model and indicated a good reliability level (Hair et al., 2011,Bashir et al., 2020).

While testing the validity, the Average Variance Extracted (AVE) of the constructs has been evaluated/assessed. It has been observed that the AVE of the respective constructs was higher than 0.50 and indicates a sufficient level of convergent validity (Mela and Kopalle, 2002).

**Figure 2:**
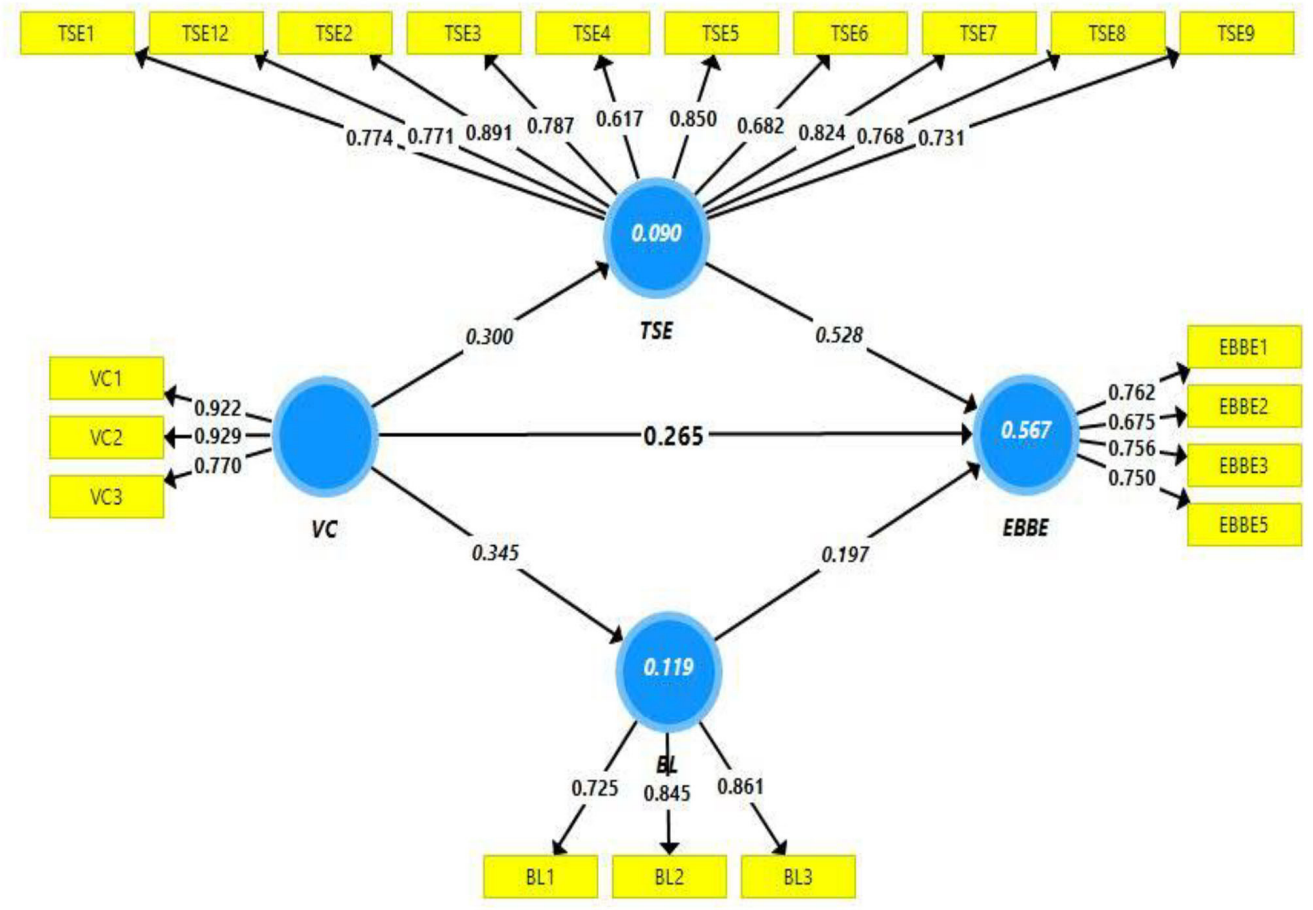
Path estimates.

While outer loadings are used as another measure to assess the convergent validity (Figure 2 and Table 2). Here, each item/indicator of scale/construct was inspected for outer loadings, and it was observed that all the indicators pose good outer loading values despite a few items. In this regard, most of the items have outer loading values above the threshold limit of 0.708. All the items of form the construct belongingness possess good outer loadings value. A similar case was observed for value consonance and no item was dropped from this construct. However, one item from the construct employee-based brand equity has been dropped due to poor outer loadings (EBBE-4). Similarly, two items from teachers' self-efficacy, (TSE-10 and TSE-11) were dropped due to weak outer loadings. However, one item with loading <0.708 (TSE-4) was retained, despite poor outer loading because the AVE of this construct was above the threshold value (>*0.5*0).

**Table 2:** Reliability and convergent validity of the study constructs.

**Construct**	**Indicator**	**BL**	**VIF**	**Cronbach's Alpha**	**rho_A**	**Composite reliability**	**AVE**
BL	BL1	0.725	1.346	0.744	0.774	0.853	0.660
	BL2	0.845	1.678				
	BL3	0.861	1.575				
EBBE	EBBE1	0.762	1.207	0.737	0.761	0.826	0.543
	EBBE2	0.675	2.052				
	EBBE3	0.756	1.401				
	EBBE5	0.750	2.308				
TSE	TSE1	0.774	2.090	0.924	0.939	0.936	0.598
	TSE12	0.771	3.980				
	TSE2	0.891	4.605				
	TSE3	0.787	3.512				
	TSE4	0.617	1.535				
	TSE5	0.850	3.159				
	TSE6	0.682	1.802				
	TSE7	0.824	5.047				
	TSE8	0.768	3.428				
	TSE9	0.731	1.961				
VC	VC1	0.922	2.487	0.855	0.939	0.908	0.769
	VC2	0.929	3.158				
	VC3	0.770	1.816				

*VC, Value consonance; EBBE, Employee based brand equity; TSE, Teacher self-efficacy; BL, Belongingness*.

While talking about discriminant validity, this study used the well-established criteria of and heterotrait-monotrait (HTMT) ratios (Hair et al., 2017). Tables 3, 4, in this regard, illustrate these two criteria. The first criteria in this regard indicate that the square root of the AVE of variables is higher than the correlations among them (Joe F Hair et al., 2011). Hence, the square root of the AVE of each construct is observed larger as compared to the correlation in the respective column and row, indicating an established Fornel-Larcker Criteria.

**Table 3:** Discriminant validity (HTMT).

**Construct**	**BL**	**EBBE**	**TSE**	**VC**
BL	-	-	-	-
EBBE	0.588	-	-	-
TSE	0.344	0.668	-	-
VC	0.419	0.596	0.312	-

*VC, Value consonance; EBBE, Employee based brand equity; TSE, Teacher self-efficacy; BL, Belongingness*.

**Table 4:** Direct, indirect, and total path estimates.

	**Beta**	**SD**	**t**	**p**
**Direct path**
BL -> EBBE	0.197	0.052	3.758	0.000
TSE -> EBBE	0.528	0.041	12.873	0.000
VC -> BL	0.345	0.047	7.293	0.000
VC -> EBBE	0.265	0.040	6.649	0.000
VC -> TSE	0.300	0.055	5.495	0.000
**Indirect path**
VC -> TSE -> EBBE	0.158	0.028	5.725	0.000
VC -> BL -> EBBE	0.068	0.023	2.970	0.003
**Total path**
VC -> EBBE	0.491	0.042	11.797	0.000

*VC, Value consonance; EBBE, Employee based brand equity; TSE, Teacher self-efficacy; BL, Belongingness*.

While HTMT has been used as another method to assess the discriminant validity. Both, liberal and conservative criteria of HTMT have been established as values of HTMT ratios in all columns are <0.90 and 0.85 (Table 4).

Similarly, coefficient of determination (R^2^) and effect size (f^2^) is also used as a measure to assess the model fitness. It was observed that effect size is good and acceptable i.e., >0.01 (Hair et al., 2006; Bashir et al., 2020). In terms of coefficient of determination, it was observed that value consonance, teachers ‘self-efficacy, and belongingness are explaining the 56% change in employee-based brand equity (Figure 2 illustrates the percentage of change). Similarly, 9% change is being observed in teacher self-efficacy due to value consonance, and 12% change is being observed in belongingness due to value consonance, thus, the coefficient of determination is satisfactory (Hair et al., 2017). While predictive relevance has been tested based onQ^2^ (Geisser, 1975), which is found satisfactory (greater than zero).

### Hypotheses Testing

Finally, hypothesis testing has been done based on structural model evaluation/assessment. In this regard, *p* and *t* statistics have been evaluated while the mediation hypothesis has been tested based on the significance of indirect paths. Table 5 illustrates hypotheses testing. The first hypothesis of this study is related to the relationship of VC → EBBE (H1). Path estimates of this relationship indicates that one unit change in value consonance will bring a 0.265 unit change in employee-based brand equity. Both p and t statistics for this path are within the acceptable range (t > 1.96 and *p* < 0.05). This state of affairs indicates that the impact of value consonance on employee-based brand equity is positive and insignificant, hence H1 is supported. Similarly, the second hypothesis of this study is related to the relationship between VC → TSE (H2). Path estimates of this relationship indicates that one unit change in value consonance will bring a 0.300 unit change in teachers' self-efficacy. Both p and t statistics for this path are within the acceptable range (t > 1.96 and *p* < 0.05). This state of affairs indicates that the impact of value consonance on self-efficacy is positive and insignificant and this state of affairs indicates that H2 is supported. The third hypothesis of this study is related to the relationship of VC → Belongingness (H3). Path estimates of this relationship indicates that one unit change in value consonance will bring a 0.345 unit change in teacher's belongingness. Both p and t statistics for this path are within the acceptable range (t > 1.96 and *p* < 0.05). This state of affairs indicates that the impact of value consonance on belongingness is positive and insignificant. Thus, H3 is supported. While testing for mediation, indirect effects have been evaluated. In this regard H4 related to VC → TSE → EBBE has been evaluated based on indirect effect, which is significant, and it can be implied that value consonance promotes teacher self-efficacy, which further shapes positive behavior in terms of employee-based brand equity. Thus, H4 is accepted. Similarly, H5 is related to VC → BL → EBBE. This indirect path is evaluated based on the significant indirect effect and it can be implied that value consonance promotes belongingness which further shapes positive behavior in terms of employee-based brand equity. Thus, H5 is accepted.

**Table 5:** Hypotheses testing.

		**Coefficient (Beta)**	**S.D**	**t**	**p**	**Status**
**Direct hypotheses**			
H1	VC → EBBE	0.265	0.040	6.649	0.000	Supported
H2	VC → TSE	0.300	0.055	5.495	0.000	Supported
H3	VC → BL	0.345	0.047	7.293	0.000	Supported
**Mediation hypotheses**			
H4	VC → TSE → EBBE	0.158	0.028	5.725	0.000	Supported
H5	VC → BL → EBBE	0.068	0.023	2.970	0.003	Supported

*VC, Value consonance; EBBE, Employee based brand equity; TSE, Teacher self-efficacy; BL, Belongingness*.

## Discussion

This research was conducted to explore the possible relationships which were not taken into consideration before. The direct relationships of teachers' value consonance were tested with employee-based brand equity, teachers' self-efficacy, and belongingness. The indirect relationships between teachers' self-efficacy and belongingness were also tested in this research between teachers' value consonance and employee-based brand equity. The results proved the worth of studying these relationships in this context of brand equity which was not much explored before in higher educational institutes. The first hypothesis was about checking the association of teachers' value consonance with employee-based brand equity, and it was accepted indicating that there was a significant association of teachers' value consonance with employee-based brand equity.

This result proved that when teachers got congruence between their own beliefs and institutional values then it could lead to developing a sense of ownership for the institute. This kind of ownership develops a sense of working hard for the betterment of the organization. This betterment ultimately leads to developing the brand image of the institute. This brand image is the goal of based brand equity in which teachers and other employees work for the internal branding which ultimately attracts the customers which are the students in the context of educational institutes. This result is also supported by the fact that branding has taken on new dimensions, and as a result of its competitive character, it has become the most in-demand commodity in businesses (Urde, 2016). According to some authors (Backhaus and Tikoo, 2004), branding is more about people than it is about objects or commodities, and in this case, involved people are the students and teachers.

Furthermore, branding was originally used to distinguish between services and things, but it has since expanded to be linked to persons, helping businesses to stand out from their competition. After going deep into the concept of branding, internal branding is the next target of the organizations which is a multi-faceted strategy for promoting an organization's brand among its employees (Backhaus and Tikoo, 2004; Aurand et al., 2005; Biswas) which are teachers and administration in this study. Similar results were obtained for the direct relationship of teachers' value consonance with teachers' self-efficacy and belongingness. These hypotheses were also accepted indicating that the shared values of teachers with their institutes develop a sense of belongingness in them along with helping them in becoming more self-efficacious.

These results could be interpreted in the understanding of the value of consonance. Before going into value consonance, one should be aware of values. Personal values related to one's interests are important factors in providing inspiration and making decisions (Schwartz). A recent study discovered that how one's principles align with the perceived values promoted by one's company has an impact on one's professional wellbeing and perseverance (Cable and Edwards, 2004). Therefore, teachers' value consonance is very important in developing self-efficacy and a sense of belongingness in them. These findings could be seen as demonstrating the importance of value concordance for teachers and that education processes cannot be limited to a technical aspect only.

We argue that instruction entails the transmission and embodiment of values, and most teachers are motivated by objectives and principles which are difficult to compromise since their activity at work entails more than just education and professional skill development. As a result, most teachers are obliged to have aims and values that are consistent with the institution's goals and values. Although this study cannot draw causal conclusions, one interesting assumption which should be examined in longitudinal studies is that value consonance promotes instructors' self-efficacy and belongingness. The mediating roles of teachers' self-efficacy and belongingness were also evaluated in this research and proved that these could mediate the relationship of teachers' value consonance in developing employee-based brand equity.

The results indicated that both teachers' self-efficacy and belongingness that fully mediated the relationship and attention toward the future would help develop based brand equity in the educational institutes. Previously, it is considered that teachers' self-efficacy is a component of social cognition theory and has been widely investigated in a range of disciplines and settings, and it has been backed up by a large body of data from several domains (Fryer et al., 2021). Due to teachers' value concordance, this study affirms teacher self-efficacy as an important psychological condition that positively impacts employee-based brand equity. The findings support Liu and Hallinger's (2018) assertion that Chinese teachers' strong teaching efficacy contributes to high levels of teacher professional learning.

It shows that teachers' self-efficacy plays an important role in strengthening the benefits of value congruence in the creation of employee-based brand equity at educational institutions where they work. Previously, it was also assumed that belongingness is a central facet of motivation and placed as a pivotal factor in the hierarchy of motivation in many studies (Debreli and Ishanova, 2019). Due to its pivotal role, it aided in the relationship of value consonance with the development of employee-based brand equity. Some of the scholars (Shaterian Mohamadi and Asadzadeh, 2011; Saroughi and Cheema, 2022) also evaluated the mediating roles of teachers' self-efficacy and sense of belonging in different contexts. This research also approved some results (Etehadi and Karatepe, 2019), which supported the mediating role of self-efficacy from a different perspective.

## Conclusion

Based on the empirical findings of this, study it can be concluded that value consonance can promote positive behaviors in the workplace. When employees feel that they share the most prevalent norms at their academic institutes, it promotes their self-efficacy. Moreover, it can also be concluded that teachers with high self-efficacy can develop based on brand equity. Similarly, based on empirical findings of this study, it can also be drawn that those employees with high-value consonance develop a sense of belongingness with their academic institutes. Further, it can also be drawn that those teachers with high self-efficacy develop self-efficacy which further promotes their positive behavior in the shape of employee-based brand equity.

## Theoretical and Practical Implications

From a theoretical perspective, this study is the first one that has tested the impact of value consonance on employee-based brand equity which is the unique contribution of this study. This study endorses the premise of social cognitive theory (Bandura, 1986, 1997) and supports the argument that self-efficacy beliefs are influenced by four sources of information, thus, we believe that value consonance becomes a source of information for teachers and they develop self-efficacy which further triggers them to shape their positive behaviors in terms of employee-based brand equity. From a practical point of view, this study posits that academic institutes should focus on building value-based cultures so that teachers could develop a sense of consonance, enabling them to develop positive behaviors in the shape of based brand equity. Moreover, such practices can promote belongingness among teachers, and they can show positive behaviors in the workplace.

## Limitations of the Study

This study has also some limitations just like other cross-sectional studies. Thus, due to its cross-sectional nature, we cannot draw a causal relationship, so future longitudinal studies should be conducted to draw the causal inference. The sample size in this study is not large enough, so in future studies, a large sample size is considered to get deeper insights. This study has considered teachers' self-efficacy as a mediating variable to predicted employee-based brand equity, while in the future, other phenomena, such as collective self-efficacy and teacher engagement, can also be taken as mediating variables. Similarly, in future studies, dimensions of employee-based brand equity, such as brand allegiance and brand endorsement, could also be tested separately. Moreover, in future studies, belief-driven favoritism and conflict can also be accounted for, while the prevalence of ethical leadership and servant leadership can also be tested as a moderating mechanism in future studies.

## Data Availability Statement

The original contributions presented in the study are included in the article/supplementary material, further inquiries can be directed to the corresponding author.

## Author Contributions

XY drafted initial file. AH collected data. YK ran analysis file. NQ revised and proof read the file. TK and WW corrected and finalized the file. All authors contributed to the article and approved the submitted version.

## Conflict of Interest

The authors declare that the research was conducted in the absence of any commercial or financial relationships that could be construed as a potential conflict of interest.

## Publisher's Note

All claims expressed in this article are solely those of the authors and do not necessarily represent those of their affiliated organizations, or those of the publisher, the editors and the reviewers. Any product that may be evaluated in this article, or claim that may be made by its manufacturer, is not guaranteed or endorsed by the publisher.
